# Crown and Bridge Work

**Published:** 1887-03

**Authors:** J. Taft


### Crown and Bridge Work.

BY J. TAFT.

(Read before the Cincinnati Odontological Society.)

This manner of substitution has within the last few years received considerable attention in the way of discussions, and more in the way of experiment and testing the various methods that have been suggested. In respect to this, like many other new things, it presents some quite practicable features, and others of less value, and still others of decidedly objectionable character. The most easily applied and valuable substitute is that of single gold crowns applied to good roots, or to roots, though diseased, that may be rendered comfortably healthy. The methods of adapting these may be readily understood by a few words of explanation.

In the first place, every one will readily understand that a first consideration is a good root, and if not in good condition at first presentation, it should be made as nearly so as possible before placing a crown upon it, though there are cases in which roots may be as efficiently treated after as before a crown is affixed. Those roots that have no discharge through the root-canals may be regarded as of this class. In almost every instance when the discharge from the diseased root is wholly through the alveolus and gum, the treatment will be more easy and efficient after a crown is applied and used in mastication; indeed, in some cases, the only treatment necessary to establish a healthy condition about a diseased root, is to give it legitimate work to do. Restoration will be attained in many cases where there is not a large accumulation of debris and foreign substance, and even here the simple removal of this and thorough washing will be all that will be required. Any discharge through the root must be arrested or diverted before a crown can be safely placed upon it. In some instances, a complete arrest is not easily affected, and a diversion of the discharge into a new channel, may be the easiest way to success. After the condition of the root has been sufficiently restored for the reception of a crown, the next step is dressing the root. If there is any part of the crown standing, at least having any considerable strength, it should be retained, at least so far as the adaptation of the crown will admit, all softened and decayed parts should be removed; the root canal filled, any remaining part of the crown should be dressed so that a band will pass over it and fit closely to the neck of the tooth, under the margin of the gum. Irregularities of the remaining portion of the natural crown need not be removed.

The root being in readiness, the crown is now to be prepared. The first step to this end is, with a fine brass wire, embrace the neck of the tooth or root, twist the free ends of the wire till the tooth is bound tightly, then slip this wire band off, without changing its shape; now take a block of hard wood, half an inch square, more or less, and three or four inches long, and trim one end till it will just fit into the wire band; this is a mandrel on which the collar or band part of the crown is to be made and formed. The collars or bands can be obtained at the dental depots; they are made of various sizes, both in respect to diameter and width, to suit the various sizes of teeth. A scale for the measurement of teeth has been published in several dental journals.

Quite a good method is for each dentist to make, or have made, an assortment of these bands for his own use, so that he will always have at hand whatever is needed for any case. The bands should be made of coin gold, rolled to No. 30, Stub's gauge. After the wooden mandrel is made, the little wire band may be cut open, which will give the exact length of the plate for the circumference of the crown band. The width of this band must correspond to the required length of the crown. The band is now formed on the wooden mandrel, jointed and soldered. The joint should be a perfect one, so that the least possible amount of solder will be required. The band is now easily adjusted to the root; it should fit tightly, requiring a few taps with the mallet and the driver to send it to its place. Its edge should pass sufficiently beneath the free margin of the gum to protect the cementum and dentine from contact with outside substances. Attention should now be given to the required length of the crown, and the band dressed accordingly. The crown cap is now to be prepared. It should be made of the same plate as the band. To form it, the crown-dies and apparatus of Dr. J. J. R. Patrick, of Illinois, afford the greatest facilities, a few moments of time only being required for shaping and fitting it. This can be adjusted upon the band while upon the root, so that the occlusion with the teeth of the opposite jaw will be accurate. The whole may now be removed from the mouth, the cap soldered to the band and finished. If great force is to be made upon the crown in masticating, fine gold solder may be flowed, filling the depressions on the concave side of the cap before it is soldered on to the band. The crown being finished, it is ready to be fixed in position. Before doing this, however, it may be placed on the root, and the occlusion observed, and if opposing cusps strike too hard at any point on the new crown, a corresponding depression may be made with the modelling pliers.

To set the crown in place, protect the root from moisture with napkins or any convenient appliance; dry as well as may be with absorbents and warm air; then fill the concavity of this part with oxyphosphate, lining the crown inside with it; then promptly place the crown on the root and put it home by firm pressure with the thumb, or drive it with the mallet, and the work is complete. This work I regard as very valuable for molars and bicuspids.

